# Untangling the Complex Associations between Socioeconomic and Demographic Characteristics and Prenatal Detection and Outcomes in Congenital Heart Disease

**DOI:** 10.3390/jcdd11050155

**Published:** 2024-05-18

**Authors:** Jennifer H. Klein, Mary T. Donofrio

**Affiliations:** Children’s National Hospital, Washington, DC 20010, USA

**Keywords:** social determinants of health, congenital heart disease, prenatal detection

## Abstract

Recent literature has established a strong foundation examining the associations between socioeconomic/demographic characteristics and outcomes for congenital heart disease. These associations are found beginning in fetal life and influence rates of prenatal detection, access to timely and appropriate delivery room and neonatal interventions, and surgical and other early childhood outcomes. This review takes a broad look at the existing literature and identifies gaps in the current body of research, particularly as it pertains to disparities in the prenatal detection of congenital heart disease within the United States. It also proposes further research and interventions to address these health disparities.

## 1. Introduction

Prenatal detection is now the gold standard by which congenital heart disease (CHD) is diagnosed and managed, beginning in fetal life. The benefits of prenatal detection are numerous and include education and psychological support for families, time for expanded testing for co-morbidities, and adequate time for preparation, including delivery planning and mobilization of medical teams to care for unstable infants with CHD. Rates of prenatal detection vary dramatically by cardiac lesion, ranging between 10 and 90% [1]. 

Despite the increased focus and attention on expanding prenatal diagnosis, significant disparities still exist in who receives a prenatal diagnosis. Overall rates of prenatal detection have not significantly improved, even since the American College of Gynecology included the outflow tracts view in their recommendation for cardiac evaluation on obstetric screening ultrasound [2]. Major health organizations, including the American Heart Association, have called for research and action to mitigate such disparities. The AHA Scientific Statement on addressing social determinants of health (SDOH) asserts that “research on SDOH factors that influence outcomes in congenital heart disease are key to guide health policy conversations and initiatives [3]”.

SDOH is a broad and loosely defined term that encompasses the non-medical factors that influence health outcomes [4]. It attempts to describe the social, economic, political, and environmental conditions in which individuals live, work, grow, and access healthcare. The primary purpose of this review is to provide an overview of the current literature on SDOH in fetal CHD within the United States, specifically its impact on prenatal detection. Secondarily, we call attention to gaps in the research and call for focused, equitable, and modifiable strategies to challenge the status quo.

## 2. SDOH and Prenatal Detection

Disparities in outcomes for CHD patients and their families begin in fetal life. This review places a particular focus on prenatal detection as the starting point for a life-long relationship with the healthcare system that is influenced not only by medical factors, but also by the SDOH at play. The life-course perspective proposes that stressors and disadvantages, even at early developmental stages, impact health outcomes throughout one’s life, underscoring how SDOH during fetal life may influence lifelong disparate outcomes. Public health phenomena such as “weathering” or “allostatic load” (defined in Table 1) of socioeconomic stressors describe the cumulative “wearing down” of one’s overall health across the lifespan, illuminating the importance of addressing disparities starting in fetal life. 

We consider prenatal disparities research to fall into five broad categories: race/ethnicity, income, health insurance status, location/rurality, or other factors. Other factors include maternal education or preferred language, for example. Research into the SDOH influences on prenatal detection largely falls into one or more of these five categories. The selected studies discussed below are outlined in Table 2.

### 2.1. Race/Ethnicity

Given the abundance of SDOH literature on disparate health outcomes by race or ethnicity across a wide variety of health measures, there is surprisingly little literature on the association of maternal race/ethnicity and prenatal diagnosis of CHD. In fact, few studies to date have looked explicitly at race or ethnicity as a social construct influence how and when a prenatal diagnosis of CHD is made.

Studies that included race/ethnicity in their analyses have found consistently lower rates of prenatal detection among Hispanic mothers. In a single-center study of infants with critical CHD, Hispanic infants were significantly less likely to have received a prenatal diagnosis, with 42% lower odds of prenatal detection after accounting for other variables, compared to other racial or ethnic groups [1]. Similarly, a study from the National Birth Defects Prevention Study (NBPDS) found Hispanic mothers were significantly less likely to have a prenatal diagnosis of CHD as compared to non-Hispanic white mothers (9.5% vs. 17.3%, respectively) [7]. A multicenter retrospective study from the Fetal Heart Society also showed Hispanic mothers were less likely to receive a prenatal diagnosis of hypoplastic left heart syndrome (HLHS) or transposition of the great arteries (TGA) as compared to non-Hispanic white mothers [13]. Several other single-center studies, while not the primary outcome of interest, found no differences in prenatal detection of CHD by mother’s race/ethnicity [9,11,16,17]. 

Race/ethnicity as an SDOH deserves increased attention. Disparities in prenatal detection among Hispanic mothers are hypothesized to be driven by language or insurance barriers, as discussed below. Additionally, selecte literature has shown a higher incidence of CHD by race/ethnicity, particularly among minorities [18,19]. However, this is particularly difficult to study, as racial designations are subject to provider bias or rely on self-reporting and change over time. Race is a social construct but nonetheless has tangible and impactful implications for disparities in healthcare in fetal CHD.

### 2.2. Income

Maternal income reflects resources available to a family and is therefore discussed as a key SDOH in the prenatal detection of CHD. However, study design has varied greatly, with some smaller studies examining individual maternal income, but most using community-level (such as ZIP code or census tract) median income as a surrogate for maternal poverty. This variation may explain the inconsistent associations between income and the prenatal detection of CHD. 

One study of ZIP code median household income found no difference between those prenatally and postnatally diagnosed, but when the same population, controlled for echocardiogram view, were required to make the diagnosis in the presence of extracardiac anomalies, maternal poverty (as determined by lower ZIP code-level median income) predicted lower rates of prenatal detection [11]. In a similar study of census tract-level median household income, mothers from census tracts with 10–20% of the population below the poverty level were less likely to undergo a prenatal ultrasound. But of those who did undergo ultrasound, there was no difference in prenatal detection by census tract poverty level [16]. Similarly, the Fetal Heart Society study of HLHS and TGA cases found no differences in the prenatal detection rate for those mothers who lived in census tracts with >20% poverty rate [13].

Contrary to the null findings described above, in a large review of over 4700 Medicaid claims the median household income of the mother’s ZIP code was significantly associated with prenatal detection of CHD, with higher median income associated with higher rates of prenatal detection [8]. However, the study population is presumed to have some level of baseline poverty, given the fact that they had filed a claim with Medicaid. Perhaps these findings better reflect the opportunities of a given neighborhood rather than the true income or poverty status of the mother. Finally, the only studies to directly examine household income were small studies in which no difference in prenatal detection of CHD by income was discovered [1,9,17]. 

These contradictory findings call for further investigation into income/poverty as an SDOH in the prenatal detection of CHD. Like race/ethnicity, there are some preliminary data that mothers living in lower-income communities are more likely to have an infant with CHD [20], and this therefore deserves further study. However, median household income is an indirect measure of the realities in which a family lives and efforts should be made to understand the true income and resources available to a family.

### 2.3. Health Insurance Status

Maternal health insurance status as a SDOH reflects both maternal poverty and issues of healthcare access. Patients insured through Medicaid are known to be lower-income and experience greater healthcare barriers as compared to their privately insured counterparts [21]. Multiple studies demonstrate an association between maternal health insurance status and lower rates of prenatal detection of CHD.

A study in 2009 found that having private insurance (versus public insurance, such as Medicaid) was a strong predictor of receiving a prenatal diagnosis of critical CHD [15]. A more recent statewide study from Arizona also found private insurance to be significantly associated with higher rates of prenatal detection, as compared to public insurance [14]. Regardless of insurance type, the presence of any health insurance during pregnancy is more likely to increase the likelihood of a prenatal diagnosis of CHD [1]. Even among studies that found no difference in prenatal detection of CHD by insurance type, public insurance was associated with a later gestational age at the time of prenatal diagnosis, as compared to those with private insurance [10,13].

The importance of insurance status is unique to the U.S. healthcare system, where insurance status is closely tied to both poverty and access to medical care. It is therefore not surprising that health insurance is associated with the adequacy and timing of the prenatal detection of CHD in the United States.

### 2.4. Location/Rurality

Maternal location is one of the most widely examined SDOHs pertaining to the prenatal detection of CHD. Location has been defined by rural versus urban address, driving distance or travel time to a tertiary care center, or the population density of a given area where the mother resides. 

Across these varying metrics, location is a strong predictor for prenatal detection of CHD [13,14]. For instance, the rate of prenatal detection in the state of Arizona was significantly lower, at only 36%, for mothers residing in rural ZIP codes, as opposed to urban ZIP codes, where the prenatal detection rate of CHD was 55% [14]. Even within the Canadian universal healthcare system, disparities have been found in rates and timings of CHD diagnosis. Specifically, rural location is associated both with lower rates of prenatal detection and with prenatal detection occurring at a later gestational age [12]. 

Location-based disparities are hypothesized to be related to healthcare quality and access. In a single-center study from Wisconsin, prenatal detection was significantly lower for those individuals living in a rural area [11]. However, those cases for which the diagnosis could be made with the four-chamber cardiac view showed no difference in rates of prenatal detection, reflecting the knowledge and ability of care providers in rural areas to make a fetal cardiac diagnosis using non-standard views. A 10-year review of CHD cases in Utah found that rural residence had no impact on whether a mother received a prenatal ultrasound, but did impact whether a prenatal diagnosis of CHD was made [16]. A 6-year review of the Society of Thoracic Surgeons (STS) Congenital Heart Surgery Database found significant variations in the rate of prenatal detection across broad regions of the United States, with east coast areas having the highest rate of prenatal detection [22]. Those studies that examined distance from a fetal cardiologist or fetal echocardiogram found no difference in rates of prenatal detection by location [1,15].

In summary, location-based SDOH is a strong candidate for interventions to improve prenatal detection across the United States. Novel tools such as geospatial analysis and technology-driven care through artificial intelligence and telemedicine may mitigate the location-based disparities that impact care for fetal CHD.

### 2.5. Other

SDOHs in the prenatal detection of CHD go beyond race, ethnicity, income, insurance, and rurality. Prior investigations have also examined maternal education, employment, preferred language, and aggregate measures of socioeconomic status, to name a few. 

Lower educational attainment was associated with less frequent prenatal detection in one study, but showed no difference in other models [7,16,17]. Primary language has also shown mixed results in its association with prenatal detection. One study found non-English-speaking patients were more likely to have a postnatal diagnosis or later-gestational-age diagnosis [10]. Another study found no difference in prenatal detection by primary language, but this same study found Hispanic mothers to be less likely to have a prenatal diagnosis [1]. Certainly, there is collinearity between ethnicity and preferred language, and thus these SDOHs are difficult to tease apart.

Several studies have used aggregate measures of socioeconomic status or social vulnerability, such as the Area Deprivation Index (ADI) or Socioeconomic Quartile (SEQ), which calculate a relative measure of socioeconomic status and opportunity based upon multiple metrics of a given neighborhood. Such metrics may include median household income, average educational attainment, employment statistics, etc. Overall, patients from disadvantaged neighborhoods, as measured by aggregate SES metrics, show lower rates or later timings of prenatal detection, including one study that found patients from census blocks in the highest socioeconomic quartile had a 62% rate of prenatal detection, as compared to those from census blocks in the lowest socioeconomic quartile, with only 35% prenatal detection (*p* < 0.001) [15]. The complexity in using these measures is that (1) aggregate measures may lose granularity in teasing apart which, if any, factors have the greatest influence on prenatal detection and (2) it is not known how to compare the many different aggregate measures of socioeconomic status that exist and are used across the literature. 

Several studies have also included maternal health indicators in their analyses of SDOH. For instance, maternal diabetes and obesity have higher incidences in communities of disadvantage [23,24]. As obesity is a known limitation in detecting CHD [7,16], it follows that such communities may experience lower rates of prenatal detection. Conversely, maternal diabetes is known to be associated with an increased risk of CHD [25,26], and therefore these patients are more carefully screened for fetal CHD. The precise interplay between nutritional access, socioeconomic opportunity, and maternal health deserves more dedicated investigation.

## 3. SDOH on Surgical and Other Early Childhood Outcomes

The impact of SDOH on surgical and other early-childhood outcomes in CHD is too broad to adequately cover in this review. It is worth noting that there is extensive research into the disparities that exist in a variety of health outcomes, including surgical mortality, medical complications, and neurodevelopmental outcomes for children living with CHD [27,28,29,30,31,32,33,34]. In a broad systematic review of the literature, SDOHs are found to be “significantly associated with adverse outcomes across the lifespan of CHD patients…[including] many of the most important and serious CHD outcomes [35].” Table 3 highlights three recent review articles on SDOH and associations with postnatal outcomes, including mortality after cardiac surgery and brain development across the lifespan.

## 4. Gaps in Research and Care

The foundation of the research described is lacking in three primary areas. First, there are no large-scale or multicenter studies with individual-level SDOH data. The primary limitations of works published thus far concern both the relatively small sample sizes and the use of community-level data (such as median household income) to extrapolate to the social circumstances of a particular patient or family. Additionally, because most of these studies only include live-birth infants with CHD for whom timing of diagnosis is retrospectively determined, cases of termination or fetal demise are not included. Fetal CHD has an increased risk of fetal demise [36] and a baseline rate of elective termination [37], and thus a significant portion of the fetal CHD population is missing from these analyses. 

Second, there is a dearth of intervention-based research or Quality Improvement initiatives to address SDOH and resulting disparities in prenatal detection. For instance, there is little prospective work on the use of interpreters for limited English proficiency families, or services that fill gaps in access to care for families in rural areas, such as leveraging technology to reach disadvantaged populations and expand prenatal care for CHD. Future studies should address not only *what* disparities exist, but also *how* to address them. An examination of global disparities in the fetal detection of CHD may illuminate how standards of fetal care, healthcare access, and the application of fetal echocardiography guidelines influence rates of prenatal detection.

Finally, the impact of disparities in prenatal detection on fetal intervention and delivery planning for fetuses with CHD should be considered. Fetal intervention, such as balloon aortic valvuloplasty for critical aortic stenosis in evolving HLHS, and delivery planning with specialized care and rapid transport to a tertiary care center, are entirely unavailable to those who lack a prenatal diagnosis. Furthermore, even a late prenatal diagnosis of CHD will affect care and may preclude a patient from receiving a fetal intervention or planning for delivery at a tertiary care center. The importance of prenatal detection for fetal intervention and delivery planning in CHD outcomes is further discussed below. 

## 5. Role of Prenatal Detection on Fetal Intervention and Delivery Planning 

Prenatal detection improves outcomes for fetuses with CHD. Infants diagnosed prenatally have less acidosis, hypoxia, and preoperative brain injury in the perinatal period [38,39,40], and better long-term neurodevelopmental outcomes [41]. Prenatal detection also allows for surveillance throughout pregnancy, thus mitigating the possibility of fetal loss, as in the case of Ebstein’s anomaly or arrhythmias which can lead to hydrops fetalis. It allows for delivery planning in cases of potential clinical deterioration, such as in the premature closure of the foramen ovale in HLHS. Additionally, prenatal diagnosis allows for consideration for intervention. Fetal intervention in evolving HLHS can potentially change the clinical course from a single ventricle palliation to that of a two-ventricle repair by balloon aortic valvuloplasty [42]. Fetal intervention of an atrial stent minimizes the need for cesarean section as well as neonatal compromise in cases of in HLHS with a restrictive atrial septum [43,44].

Delivery planning for the high-risk neonate is another substantial benefit of prenatal diagnosis. Prenatal detection allows for changes in the delivery plan to be proximate to a tertiary care center, which has shown benefits in the survival of CHD [45]. It also allows for the preparation of families and medical teams in the highest-risk patients where immediate stabilization is necessary, such as in HLHS or TGA with a restrictive atrial septum, interrupted or hypoplastic aortic arch, or total anomalous pulmonary venous return. The prenatal detection of these high-risk lesions has led to improved oxygenation and faster time to care [38,46]. 

Therefore, any disparities that exist in the prenatal detection of CHD can be extrapolated to disparities in fetal interventions, delivery planning and subsequent outcomes for these high-risk patients. To our knowledge, there are no studies that examine the influence of SDOH on fetal intervention or delivery planning. In fact, the demographics of those receiving a fetal intervention are not reported in the International Fetal Cardiac Intervention Registry publication of patients who underwent fetal aortic valvuloplasty [47]. Similarly, the literature on delivery planning has not investigated SDOHs such as health insurance, income, and distance from tertiary care center, which certainly influence ability to transfer delivery care. In summary, fetal interventions, which are only offered at a few centers, and modifications of delivery plans require social and economic resources that are not uniformly available, thus underscoring the need for SDOH research in these critical and expanding areas of fetal cardiac care.

## 6. Conclusions

This review identifies at-risk populations, notably those living in rural areas, with public health insurance, or of Hispanic ethnicity, who may experience worse fetal and neonatal outcomes secondary to missed or late prenatal diagnoses of CHD. The multiple benefits of prenatal CHD diagnosis are mitigated by disparities that exist in prenatal detection as driven by SDOH. Notably, the various SDOH metrics do not exist in isolation. While they are presented as distinct factors, they are better conceptualized as broad and nuanced non-medical factors that influence and interact with one another. SDOHs reveal an individual’s perception of the need for prenatal screening and their ability to access such care. This encompasses everything from economic resources to community support. In summary, women do not seek prenatal care in a vacuum. Rather, they navigate a complex system of insurance coverage, literacy, lost wages from time away from work, and childcare for other children. As providers, we must understand and appreciate these complexities and challenges.

In this review, we have not untangled, but only begun to pull apart, the complex associations between social determinants of health and the prenatal detection of CHD. Understanding and addressing SDOH is an ever-more important aspect of the skills and responsibilities demanded of fetal cardiac care providers. Education, research, and partnership with obstetricians and community health organizations should be a focus in the coming decades. To best care for the families and children diagnosed with CHD, we must understand the lives and realities in which our patients live, work, and seek medical care. This starts with prenatal detection and continues across the lifespan.

## Figures and Tables

**Table 1:** Public health terms.

Term	Definition
Social Determinants of Health (SDOH)	The non-medical factors, such as social, economic, political, and environmental conditions, in which individuals live, work, and grow, that in turn impact health outcomes [4]
Life-course perspective	The understanding that sequential events, stressors, and other SDOH factors influence health outcomes across the lifespan
Allostatic load	The cumulative and repetitive burden of stressors that impacts health outcomes [5]
Weathering	The effect of cumulative and repetitive stressors that “wear down” the health and well-being of marginalized populations [6]

**Table 2:** Summary of selected SDOH articles on prenatal detection of CHD.

Author	Title	Year, Publication	Study Type	SDOH Investigated
Ailes et al. [7]	Prenatal diagnosis of non-syndromic congenital heart defects	2014, Prenatal Diagnosis	National database, retrospective. (n = 7299)	Race, education
Campbell et al. [8]	Socioeconomic barriers to prenatal diagnosis of critical congenital heart disease	2020, Prenatal Diagnosis	National, retrospective. (n = 4702)	Race, income, sonographer location quotient
Davtyan et al. [1]	Prenatal diagnosis rate of critical congenital heart disease remains inadequate with significant racial/ethnic and socioeconomic disparities and technical barriers	2023, Pediatric Cardiology	Single center, retrospective. (n = 339)	Race, language, insurance, income, distance from care, ADI
Friedberg et al. [9]	Prenatal detection of congenital heart disease	2009, Journal of Pediatrics	Multicenter, prospective. (n = 336)	Race, income, education, employment, insurance
Gianelle et al. [10]	The impact of neighborhood socioeconomic status, race and ethnicity, and language on prenatal diagnosis of CHD	2023, Pediatric Cardiology	Single-center, retrospective. (n = 163)	Race/ethnicity, language, SEQ
Hill et al. [11]	Disparities in the prenatal detection of critical congenital heart disease	2015, Prenatal Diagnosis	Single center, retrospective. (n = 535)	Race, insurance, income, population density
Kaur et al. [12]	Impact of rural residence and low socioeconomic status on rate and timing of prenatal detection of major congenital heart disease in a jurisdiction of universal health coverage	2022, Ultrasound in Obstetrics and Gynecology	Canadian province, retrospective. (n = 1405)	Chan Index SES, distance from tertiary care center, IOR, ROR
Krishnan et al. [13]	Impact of socioeconomic status, race and ethnicity, and geography on prenatal detection of Hypoplastic Left Heart Syndrome and Transposition of the Great Arteries	2021, Circulation	Multicenter, retrospective. (n = 1862)	Race, insurance, residence location, SEQ
Mattia et al. [14]	Prenatal detection of congenital heart disease: recent experience across the state of Arizona	2023, Prenatal Diagnosis	Single-center, retrospective. (n = 1137)	Race/ethnicity, insurance, rural address
Peiris et al. [15]	Association of socioeconomic position and medical insurance with fetal diagnosis of critical congenital heart disease	2009, Circulation: Cardiovascular Quality and Outcomes	Single-center, retrospective. (n = 444)	Race, insurance, driving distance to care, SEQ
Pinto et al. [16]	Barriers to prenatal detection of congenital heart disease: a population-based study	2012, Ultrasound in Obstetrics and Gynecology	Statewide, retrospective. (n = 1474)	Race, education, income, travel time, rural location
Sekar et al. [17]	Diagnosis of congenital heart disease in an era of universal prenatal ultrasound screening in southwest Ohio	2015, Cardiology in the Young	Single-center, prospective. (n = 100)	Race, education, income, insurance

ADI: Area Deprivation Index. SEQ: Socioeconomic Quartile. SES: socioeconomic status. IOR: Index of Remoteness. ROR: Remoteness of Residence.

**Table 3:** Review articles on SDOH and surgical and early childhood outcomes.

Author	Title	Year, Publication	Primary Outcome(s)
Davey et al. [35]	Social determinants of health and outcomes for children and adults with congenital heart disease: A systematic review	2021; Pediatric Research	Infant mortality, post-surgical outcomes, healthcare access, neurodevelopmental outcomes, quality of life
Jackson et al. [33]	Structural racism, social determinants of health, and provider bias: Impact on brain development in critical congenital heart disease	2023; *Canadian Journal of Cardiology*	Brain development
Tran et al. [32]	Social determinants of disparities in mortality outcomes in congenital heart disease: A systematic review and meta analysis	2022; *Frontiers in Cardiovascular Medicine*	Mortality

## Data Availability

No new data were created or analyzed in this study. Data sharing is not applicable to this article.
